# m6A modification promotes miR-133a repression during cardiac development and hypertrophy via IGF2BP2

**DOI:** 10.1038/s41420-021-00552-7

**Published:** 2021-06-26

**Authors:** Benheng Qian, Ping Wang, Donghong Zhang, Lianpin Wu

**Affiliations:** 1 Department of Cardiology, The Second Affiliated Hospital of Wenzhou Medical University, 109 Xueyuan Road, Wenzhou, 325027 Zhejiang PR China; 2 School of Medical Imaging, Tianjin Medical University, Tianjin, 300203 China

**Keywords:** Cell proliferation, Cardiac hypertrophy, Gene silencing

## Abstract

Both N6-methyladenosine (m6A) RNA modification and microRNAs (miRNAs) are common regulatory mechanisms for gene post-transcription by modulating mRNA stability and translation. They also share the same 3′-untranslated regions (UTRs) regions for their target gene. However, little is known about their potential interaction in cell development and biology. Here, we aimed to investigate how m6A regulates the specific miRNA repression during cardiac development and hypertrophy. Our multiple lines of bioinformatic and molecular biological evidence have shown that m6A modification on cardiac miR-133a target sequence promotes miR-133a repressive effect via AGO2-IGF2BP2 (Argonaute 2—Insulin-like growth factor 2 mRNA binding protein 2) complex. Among 139 cardiac miRNAs, only the seed sequence of miR-133a was inversely complement to m6A consensus motif “GGACH” by sequence alignment analysis. Immunofluorescence staining, luciferase reporter, and m6A-RIP (RNA immunoprecipitation) assays revealed that m6A modification facilitated miR-133a binding to and repressing their targets. The inhibition of the miR-133a on cardiac proliferation and hypertrophy could be prevented by silencing of *Fto* (FTO alpha-ketoglutarate dependent dioxygenase) which induced m6A modification. IGF2BP2, an m6A binding protein, physically interacted with AGO2 and increased more miR-133a accumulation on its target site, which was modified by m6A. In conclusion, our study revealed a novel and precise regulatory mechanism that the m6A modification promoted the repression of specific miRNA during heart development and hypertrophy. Targeting m6A modification might provide a strategy to repair hypertrophic gene expression induced by miR-133a.

## Introduction

Cardiac myocytes undergoing hypertrophy responds to the development and stress by extracellular stimuli, including cytokines or pressure overload [1]. These in turn induce the reprogramming of cardiac gene expression and the activation of ‘fetal’ cardiac genes [2]. However, persistent cardiac hypertrophy induced by pathological conditions may eventually lead to ventricular dilation, heart failure, or sudden death. Although increased studies have investigated the molecular and genetic mechanisms underlying cardiac development and remodeling, additional mechanisms remain to be elucidated. Epigenetic processes, including non-coding RNAs [3, 4], N6-methyladenosine (m6A) RNA [5], chromatin and histone proteins [6] as well as DNA methylation [7], have been implicated as modulators of cardiac gene expression in heat development and disease.

Of note, cardiac miRNAs (miR-1, miR-133a, miR-208a/b, miR-499, etc.) are specific and highly expressed in the myocardium. They play key roles in cardiogenesis, heart function, and pathology. Particularly, miR-133a is essential for proper heart development and cardiac differentiation from embryonic stem cells (ESCs) [8]. MiR-133a represses cardiac hypertrophy and hypoxia-induced apoptosis [9], but promotes regeneration and cardiac programming in post-myocardial infarction (MI) [4]. While miR-499 regulates the late cardiogenesis and deregulation in pathogenesis of heart hypertrophy [10]. Similar to miR-133a, miR-499 could promote the generation of cardiomyocytes in vitro [11] and in vivo [12] via reprogramming strategies that provide new promise for recovering injured cardiac tissue after MI. In addition, N6-methyladenosine (m6A) RNA modification is the most prevalent modifications of messenger RNAs (mRNAs). m6A is often enriched in the 3′ untranslated regions (UTRs) and the stop codons of mRNAs with a consensus sequence of RRACH [13]. The biological importance of m6A is dependent on its binding proteins (that is, readers) to regulate mRNA splicing, translation, decay, or stability [14].

Interestingly, both m6As and miRNAs could bind to 3’UTRs and regulate mRNA stability [15]. Recent high throughout sequencing has indicated that m6A peaks are often enriched at miRNA target sites [13, 16]. However, the interaction of m6A and cardiac miRNAs during heart development and hypertrophy remains unclear. Our current study identified cardiac miR-133a targets were enriched by m6A modification. IGF2BP2, as the key m6A reader precipitated with the RISC complexes—AGO2, enhances the miR-133a binding and repressing their targets during heart development and hypertrophy.

## Results

### m6A modification associates cardiac miR-133a binding to their targets

**Fig. 1:**
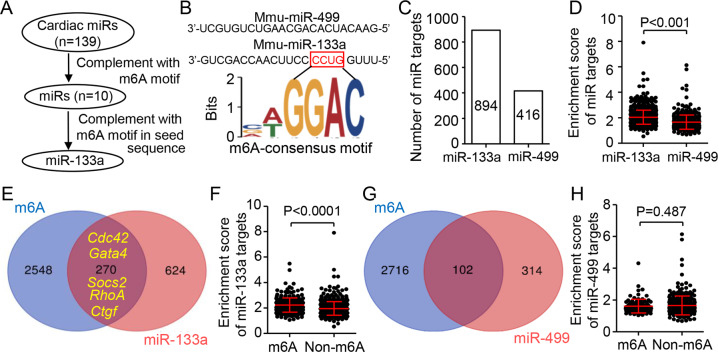
m6A modification promotes cardiac miR-133a enrichment to its targets. **A** Schematic of screening cardiac miRNAs and identifying miR-133a targets with m6A modification. **B** m6A consensus motif “GGACH” was inversely complement to the seed region of miR-133a, but not to miR-499. **C**, **D** Comparison showing the number and enrichment score of miR-133a or miR-499 targets. The data are obtained from the RISCome RNA-sequencing (Ago2) pull-down on ventricular tissues of 8-week-old male FVB/N mice. **E** Venn diagram showing the overlap of m6A-modified genes and miR-133a target genes. **F** The difference of the enrichment score of miR-133a target genes with or without m6A modification. **G** Venn diagram showing the overlap of m6A-modified genes and miR-499 target genes. **H** Overall enrichment scores of miR-499 target genes with or without m6A modification. *P* values were calculated by unpaired two-tailed Student’s *t test* (**D**, **F**, and **H**).

Both m6A modification peaks and miRNA target sites are frequently enriched in the 3’UTR region [15]. We first sought to investigate whether m6As are associated with the binding of cardiac miRNAs, which are specially and abundantly expressed in the myocardium. By sequence alignment analysis, we found 10 of 139 mouse cardiac miRNAs [17] inverse containing the m6A consensus motif “RRACH”. Especially, the strongest “GGACH” consensus motif was only inversely complement to the seed region of miR-133a, an essential regulator for cardiac development and disease [18] (Fig. 1A, B and Extend excel S1). Then, we queried the specific targets of miR-133a and miR-499 (without m6A consensus motif) in mouse hearts in published RNA-induced silencing complexes (RISC) RNA-sequencing database [19]. Overall, there were high levels of the target gene number and enrichment score of miR-133a compared to those of miR-499 (Fig. 1C, D and Extend excel S2, 3). To explore the enrichment of m6A modification on miRNA targets, we screened out genes with m6A modification from the reported m6A-RIP-sequence in adult mouse heart [13], and from them we further identified 2,818 genes containing the m6A consensus motif “GGACH” in their 3’UTR regions. The overlapping map showed that 30.20% (270/894) of miR-133a targets could be modified by m6A (Fig. 1F). Of them, *Cdc42* [20], *Gata4* [21], *Socs2* [22], *RhoA* [23] and *Ctgf* [24, 25], as the key targets of miR-133a, play a critical role in heart development and hypertrophy [26]. Interestingly, the enrichment scores of miR-133a on the genes co-targeted by m6A and miR-133a were significantly higher than those of genes targeted by miR-133a only (Fig. 1F). However, comparing with miR-133a, there was few miR-499 targets with m6A modification (24.52%, 102/416) and no difference between miR-499 enriched scores with m6A modification or not (Fig. 1G, H). Together, our results suggested the m6A modification associates with the enrichment of cardiac miR-133a on their targets.

### m6A modification strengths the miR-133a repression in heart development

**Fig. 2:**
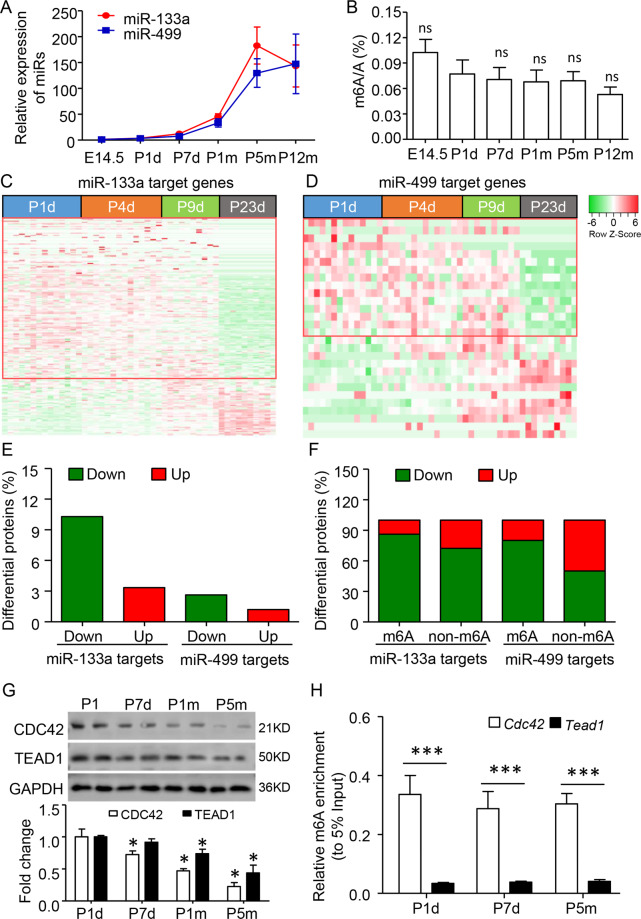
m6A modification promotes miR-133a repression in heart development. **A** Developmental expression profiles of miR-133a and miR-499. The expression changes were calculated by comparing with E14.5 data. **B** Developmental global m6A modifcation levels, as determined by ELISA analysis. ns, no significant as compared to P1d. **C**, **D** Heatmap showing the developmental changes of miR-133a and miR-499 target protein expression from the published proteomics database. The ventricular tissues were collected from the postnatal day 1 (P1) to P23 of the C57BL/6 mouse. **E** The percentage of the down- and upregulated proteins (Ratio of P23d to P1d) by miR-133a and miR-499. **F** The proportions of down- and upregulated genes targeted by two miRs with or without m6A modification. **G** Quantitative western blot analysis showed that the decreased CDC42 and TEAD1, two miR-133a target proteins in the developing ventricle from P1d to postnatal 5 months (P5m). Error bars, SD (*n* = 4–5/stage), *P < 0.05 as compared to P1d. **H** Enrichment of m6A modification at *Cdc42* and *Tead1* was analyzed by m6A-RNA immunoprecipitated-qPCR method. Data are mean ± SD (*n* = 4–6 per stage). *P < 0.05; ***P < 0.001 was calculated using the one-way ANOVA followed by Tukey’s test.

Both miR-133a and miR-499 are closely associated with cardiac differentiation [8, 27]. Consistent with previous studies, we found very low expression of miR-133a and miR-499 in embryonic day 14.5 (E14.5), but a significant increase of two miRs since postnatal day 1 (P1) and persistent to 5 months (Fig. 2A). Reversely, the ELISA assay showed that m6A levels were consistent after birth (Fig. 2B). The global proteomic profiles of miR-133a and miR-499 targeted genes during the postnatal stages were analyzed in C57BL/6 mice hearts [28]. Reverse to the developmental increases of two miRs, the ratios of their downregulated target (P23 to P1) to upregulated target were increased about 3.06-fold and 2.19-fold, respectively. Of note, there was 3.91-fold of miR-133a downregulated-target than that of miR-499 (Fig. 2C–F). The downregulated ratio of miR-133a (86.21% vs 72.34%) or miR-499 (80.00% vs 50.00%) targets with m6A modification were higher than that of without (Fig. 2F). Moreover, we re-analyzed the transcriptome profile during FVB mice development [19]. Similar to protein levels of two miRs targets, the ratio of downregulated-target to upregulated-target of miR-133a was consistently higher than that of miR-499 from E12.5, to P1 week, 4 weeks, and 5 months (Fig. S1A–C). There was a more than 2-fold increase of miR-133a targets with m6A modification compared with that of without in each stage (Fig. S1D). For example, both *Cdc42* (with m6A modification) and *Tead1* (without m6A modification) were the miR-133a targets and closely related to heart development. We found a rapid decrease of CDC42 protein comparing with TEAD1 in developing heart from P1 day to 5 months (Fig. 2G). The reason might be related to the high level of m6A modification on *Cdc42* mRNA, but not *Tead1*, during P1 day to 5 months (Fig. 2H), which enhances the miR-133a binding and repressing on its targets.

### m6A modification facilitates miR-133a binding to its targets and preventing the cardiac proliferation

**Fig. 3:**
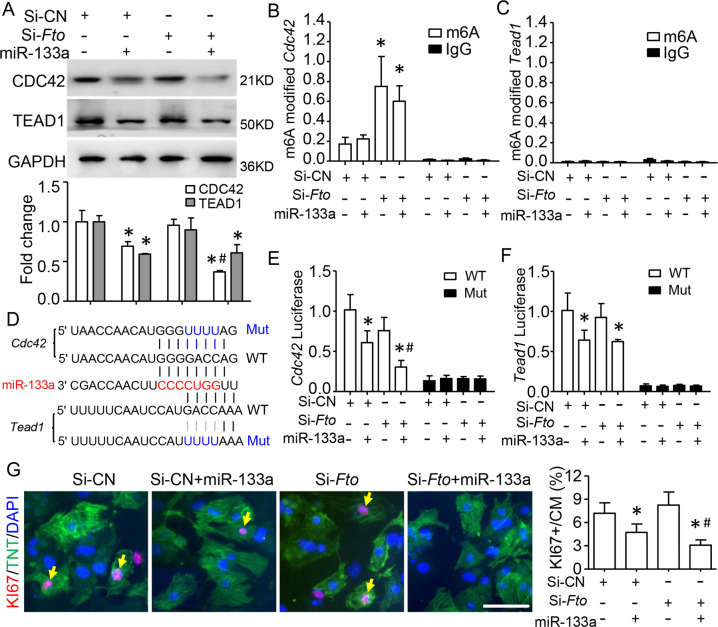
m6A modification facilities miR-133a binding and preventing cardiac proliferation. Primary cultured mice neonate cardiomyocyte (CM) was transfected with miR-133a and/or si-*Fto*. **A** Quantitative western blot analysis showed that the fold changes of CDC42 and TEAD1 protein. GAPDH served as the loading and normalization control for the quantification. **B**, **C** m6A-RIP-qPCR analyses showing m6A modification occupancy at the *Cdc42, but not* *Tead1* mRNA in CMs. The enrichment of m6A was compared with 5% input. **D** Schematic showing the binding sequences (red font) of miR-133a on *Cdc42* and *Tead1* 3’UTR region. The mutation (Mut) sites were generated as blue fonts. **E**, **F** Luciferase reporter analysis demonstrating Fto knockdown reduced the activities of *Cdc42*, but not *Tead1* by miR-133a transfection. **G** Co-immunofluorescence staining of TNT (green) and KI67 (red) confirming that the proliferated CMs was decreased by miR-133a, and continued decreased when combined with *Fto*-knockdown. Scale bar=50 μm. Data are mean ± SD (*n* = 4 per group). *p < 0.05 via Si-CN, ^#^*p* < 0.05 via Si-CN and miR-133a, was determined by one-way ANOVA followed by Tukey’s test in (**B**, **E**, **F**, and **G**).

miR-133a was reported as the regulator for cardiomyocyte (CM) development and proliferation [29]. We explored whether the m6A modification could facilitate miR-133a binding and prevent cardiac proliferation. As a previous study [30], we found FTO (FTO Alpha-Ketoglutarate Dependent Dioxygenase), a demethylase that was the most expressed m6A regulators, increased in the developed heart (Fig. S2A, B), suggesting that FTO is the major m6A regulator during heart development. Then, we isolated the primary neonatal CMs and silenced *Fto* expression by transfection of si-*Fto*. Interestingly, *Fto* knockdown could enhance the downregulation of CDC42 by overexpression of miR-133a but does not affect the decrease of TEAD1 (Fig. 3A). As expected, elevated m6A modifications were found globally, including *Cdc42*, but not *Tead1* mRNA by silencing *Fto* (Fig. 3B, C and Fig. S3A, B). However, silencing *Fto* did not affect the increase of CDC42 protein by transfected with miR-133a inhibitor (Fig. S4).

To detect if the binding of miR-133a on the *Cdc42* and *Tead1* 3’UTR could be regulated by m6A modification, we generated the wildtype and mutants of miR-133a binding sites and respectively constructed them into a pmirGLO vector for luciferase report assay (Fig. 3D). Interestingly, the reporter activities of *Cdc42*, but not *Tead1*, were significantly decreased in CMs with deletion of *Fto* by miR-133a transfection. However, this effect was disappeared by mutation of the miR-133a target site (Fig. 3E, F). Functionally, the decrease of proliferated CMs (KI67 positive) by miR-133a transfection was reduced again with *Fto* knockdown (Fig. 3G). Silencing *Fto* alone did not affect CM proliferation (Fig. 3G). Therefore, m6A modification promotes the miR-133a binding to its targets and preventing cardiac proliferation.

### m6A modification enhances miR-133a repression in cardiac hypertrophy

**Fig. 4:**
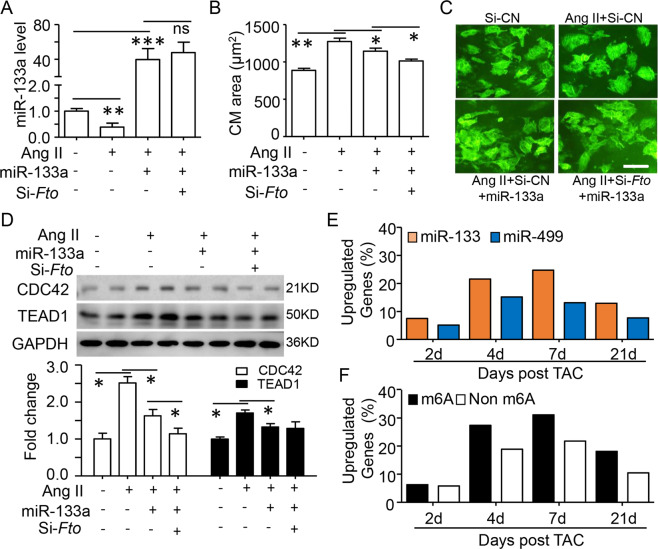
m6A modification promotes miR-133a binding in cardiac hypertrophy. Primary cultured mice neonate cardiomyocyte (CM) was transfected with miR-133a and/or si-*Fto* for 24 h and then treated by Angiotensin II (Ang II, 1 μM) for another 24 h. **A** RT-qPCR assay for miR-133a regulation in treated CMs. U6 mRNA as the intern control. **B**, **C** TNT immunofluorescence staining was performed to determine CMs size. Quantification (**B**) and representative images (**C**) of cell size from 100 CMs in each group are shown (Scale bar=50 μm). **D** Representative western blotting (up) and quantification (down) showing the changes in CDC42 and TEAD1 protein in treated CMs. **E** The percentages of the upregulated (TAC relative to Sham group) miR-133a and miR-499 targets after TAC surgery from the database of GEO (GSE101977). **F** The percentages of the upregulated miR-133a targets with or without m6A modification. Data are mean ± SD (*n* = 4 per group). *p < 0.05; ** *P* < 0.01 and ***P < 0.001 was determined by one-way ANOVA followed by Tukey’s test in (**A**, **B**, and **D**).

Except for cardiac proliferation and differentiation, miR-133a also controls cardiac hypertrophy [20, 31]. The potential role of m6A modification in this cardiac modeling was investigated by Angiotensin II (Ang II) treated neonatal CMs. As expected, Ang II could reduce miR-133a expression and increase CM size, this effect could be prevented by overexpression of miR-133a (Fig. 4A, B). Notably, silencing *Fto* strengthened the reduction of CMs size by miR-133a overexpression, but did not change the miR-133a level (Fig. 4A–C). A similar pattern was found for the regulation of CDC42, but not FTO (Fig. 4D). Consistent with the decrease of miR-133a in the hypertrophic heart, more increasing of miR-133a targets at mRNA and protein levels were found in the whole stages of TAC (thoracic aortic constriction)-induced hypertrophic hearts compared with that of miR-499 (Fig. 4E, S5). Interestingly, more ratios of upregulated (TAC to Sham group) miR-133a targets with m6A modification has been found since 4-day after TAC model introduction (Fig. 4F).

### m6A promotes the effect of miR-133a repression via IGF2BP2

**Fig. 5:**
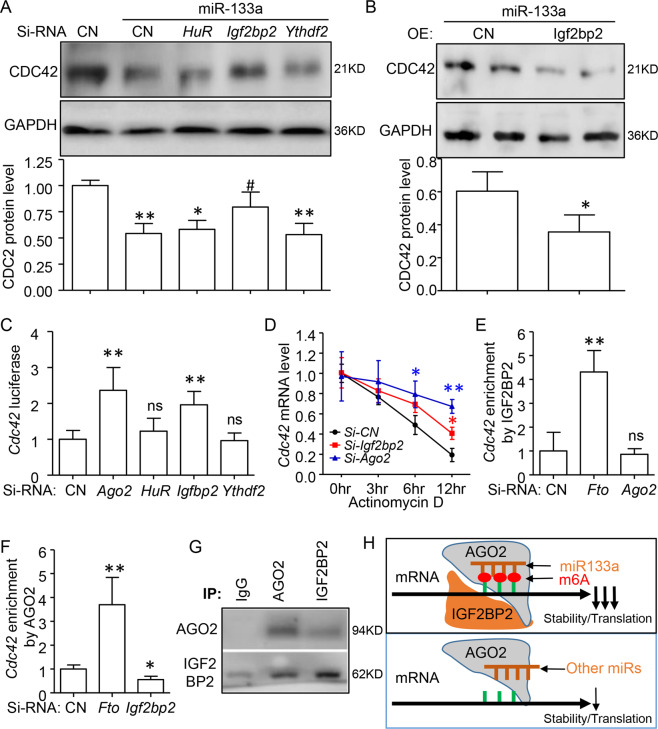
m6A modification promotes the miR-133a repression on its targets via IGF2BP2. Neonatal mouse cardiomyocytes (CMs) were transfected with miR-133a and Si-RNAs. **A** Representative western blotting (up) and quantification (down) showing the miR-133a repression on CDC42 protein level could be reversed by *Igf2bp2*, but neither *HuR* nor *Ythdf2* knockdown in CMs. **B** Western blotting assay indicating overexpression of IGF2BP2 could further decrease CDC42 with miR-133a transfection. **C** Luciferase reporter activities were quantitated in CMs and indicated miR-133a activity could increase by knockdown of *Ago2* or I*gf2bp2*, but neither *HuR* nor *Ythdf2*. **D** Inhibiting *Cdc42* mRNA decay by silencing *Ago2* or *Igf2bp2* in Actinomycin D treated CM cells. **E** RNA immunoprecipitated-qRT-PCR assay using IGF2BP2 antibody suggested that the enrichment of IGF2BP2 on *Cdc42* mRNA was increased by *Fto* knockdown, but had no effect by *Ago2* silencing. **F** AGO2-RNA immunoprecipitated-qRT-PCR assay showing the enrichment of AGO2 on *Cdc42* was increased by *Fto* knockdown but decreased by *Igf2bp2* knockdown. **G** Co-immunoprecipitation and western blotting showing the interaction of AGO2 and IGF2BP2 in CMs cells, representative of three independent experiments. **H** Carton model of IGF2BP2 binding to m6A-modified site promotes the miR-133a-AGO2-RISC complex accumulation on its targets, which would enhance the decreasing of their stability and translation. Data are mean ± SD (*n* = 4 per group). *p < 0.05; ** *P* < 0.01 was determined by one-way ANOVA followed by Tukey’s test in (**A**, **C**, **E**, and **F**). *p < 0.05 was detected by Student’s *t test* in (**B**).

Recent studies have indicated that m6A binding proteins, such as YTHDF2, HuR, and IGF2BPs regulate the m6A-modified mRNA expression [14]. Because IGF2BP2 is the major isoform in heart tissue [32] and directly binds to various mRNAs in cardiomyocytes [33], we then would like to identify whether IGF2BP2 involved in m6A and miR-133a interaction. As displayed in Fig. 5A, the western blot assay indicated that only knockdown *Igf2bp2* could reverse the repression of CDC42 by miR-133a overexpress in neonatal CMs. Overexpression of IGF2BP2 further decreased CDC42 protein expression (Fig. 5B). Similar to *Ago2*, a key compound of RISC, silencing of *Igf2bp2*, but not *HuR* and *Ythdf2* induced the luciferase activities and the stabilities of *Cdc42* mRNA (Fig. 5C, D). Importantly, the Venn diagram of IGF2BP2-RIP-ChIP data and miR targets showed a significantly higher proportion of IGF2BP2-bound genes by miR-133a targeted (22.71%) compared with that of miR-499 (12.02%) (Fig. S6A, B). IGF2BP2 preferred to bind miR-133a target genes with m6A modification (including CDC42) (Fig. S6C). Moreover, miR-133a had the highest enrichment scores on the co-binding genes by IGF2BP2 and m6A, when comparing with that of individual or non-binding genes (Fig. S6D, and Extend excel S4). The above observation suggested that IGF2BP2 facilitates the binding of miR-133a, but not miR-499 on its targets, especially for those modified by m6A.

To test whether IGF2BP2 interferes with the RISC-association on *Cdc42* mRNA in CMs, as previously proposed for other target mRNAs [34], an AGO2-RIP study was performed and showed that *Fto* knockdown could increase the enrichment of AGO2 on *Cdc42* mRNA, while *Igf2bp2* knockdown decreased this enrichment. Reversely, IGF2BP2 enrichment on the *Cdc42* gene was also enhanced by *Fto* knockdown, but not affected by *Ago2* silence (Fig. 5E, F). We next determined whether IGF2BP2 physically interacted with AGO2. Co-immunoprecipitate (Co-IP) assay showed IGF2BP2 antibodies interacting with AGO2. Meanwhile, AGO2 antibodies also immunoprecipitated with IGF2BP2 (Fig. 5G). In conclusion, these findings indicated that IGF2BP2 binding to m6A-modified site could promote miR-133a-AGO2-RISC complex accumulation on its targets and enhance the effect of miR-133a repression (Fig. 5H).

## Discussion

Increasing lines of evidence have shown that m6A modification plays a pivotal physiological and pathology function in regulating mRNA editing, transport, decay, stability, and translation [14]. However, the basic features of m6A modification, such as m6A regulation and binding have been characterized with cell-type-dependent or gene-specific [13]. Here, our study found a novel and precise regulatory mechanism of m6A modification on mRNA expression, which enhances the specific miRNA binding and repression during heart development and hypertrophy. Cardiac miR-133a has more facility to enrichment its targets with the m6A motif than without. m6A modification promotes the affection of miR-133a repression in cardiac proliferation and hypertrophic response. The m6A binding protein IGF2BP2 could increase the enrichment of the RISC-AGO2-miR-133a complex on its targets. These results have shown that m6A modification may indirectly affect physiological heart function through regulating miRNA biological processes.

Both m6A modification and miRNAs are frequently observed within 3’UTRs, act as the important post-transcriptional regulators in eukaryotes. Indeed, 80% of m6A-modified transcripts have at least one miRNA binding site and nearly all the m6A peaks (>96%) could pair with miRNAs, which is indicated from a recent m6A epitranscriptomic landscape across most human and mouse tissues [13]. Notably, the strongest “GGACH” consensus m6A motif was inversely complementary to the seed region of specific miRNAs. But only 14% of miRNAs were stably expressed in all tissues that could pair with the m6A motif. Similarly, we identified 10 of 139 cardiac miRNAs target sites have the specific m6A consensus motif “GGACH”. And only miR-133a seed sequence binding sites could fully match and functions related to the “GGACH” motif. Thus, tissue-specific miRNAs are inversely complementary to the m6A motif.

The interaction between m6A modification and miRNAs was discussed by limited studies [13, 34, 35]. Functionally, miRNAs or their biogenesis Dicer could alter the global and specific m6A abundance by mediating the accumulation of METTL3 (a major m6A methyltransferase) to mRNAs. m6A modification on those encoding developmental regulators could block HuR binding, which increasing miRNA destabilizing their expressions in mESCs [36, 37]. Reversely, we found that m6A modification enhanced the recruitment of miR-133a-RISC-AGO2 to its targets in heart development. And the targets with m6A modification showed the strict repression by miR-133a and responded to cardiac proliferation and hypertrophy, suggesting the critical role of m6A and miR-133a interaction during heart physiology and pathology.

The current three m6A binding proteins, including YTHDF2, HuR, and IGF2BPs were reported relative to the m6A-modified mRNA stability or decay [14]. HuR could prevent miRNA binding and destabilize mRNA expression with no m6A modification. IGF2BPs proteins were related to enhancing mRNA stability and translation in an m6A-dependent manner [38]. Meanwhile, IGF2BPs control mRNA turnover largely by modulating the miRNA-dependent regulation of their target transcripts [35, 39–41]. IGF2BP1 could recognize m6A modification and impair the miRNA-dependent downregulation of SRF expression, resulting in tumor cell growth and invasion [35]. IGFBP2 is the major isoform in heart tissue after birth. Consistent with IGF2BP2 increases with the miRNA-directed inhibition of effector expression in liver tissue [34], our data indicated that IGF2BP2, neither HuR nor YTHDF2, could binding to m6A modification and enhance the miR-133a-RISC-AGO2 binding to its targets, which contribute to heart development and hypertrophy. Furthermore, IGF2BP2 also could interfere with miRNA repression in some cancer cells including glioblastoma, colorectal, and thyroid cancer [41–43]. There evidence further confirmed that cell-and gene-specific miRNA and IGF2BP2 interaction should be characterized for cell biology and function.

Cardiac miR-133a has been proved to require heart development and its aberrant expression in local heart and circulation also involves cardiac disorders, such as cardiac hypertrophy and heart failure [12, 18, 20, 24, 29]. Thus, miR-133a has been recognized as a potential diagnostic and therapeutic tool for cardiac disorders. Our current study highlights the regulatory roles of miR-133a by an m6A modification dependent during cardiac development and hypertrophy. m6A modification on miR-133a target sites promotes miR-133a binding and repression. miR-133a related cardiac proliferation and hypertrophy could be altered by mediating m6A level and its binding protein, IGF2BP2. Therefore, our study uncovers a precise mechanism of m6A modification and miRNA repression in cardiac development and hypertrophy. Targeting IGF2BP2 and m6A complex could provide a strategy to impair hypertrophic gene expression induced by miR-133a.

## Methods and materials

### Database access and analysis

Transcriptome-wide m6A profiling in mouse heart tissue was downloaded from *Liu*’s study by m6A-RIP-seq (RNA immunoprecipitation followed by high-throughput sequencing) [13]. We identified 2,818 genes containing the m6A consensus motif in their stop codon and 3’UTR region. miR-133a and miR-499 target genes and their enrichment score were obtained from the published cardiac RISC complexes immunoprecipitation and sequencing [19]. IGF2BP2 protein bound genes in cardiomyocytes were gotten from IGF2BP2-RIP array [33]. miRs target genes were classified into m6A and miR-133a co-regulated genes and miR-133a only regulated genes (Supplementary Table S1). The global proteomics data of mouse heart development were accessed from *Talman*’s report [28]. Expression profiling by the array for cardiac development based on FVB mice was obtained from Gene Expression Omnibus (GEO: GSE75). We used the global gene expression (GEO: GSE101977) of cardiac hypertrophic C57BL/6 mice which were introduced TAC surgery [44].

### Cardiomyocyte isolation, culture, and transfection

Neonatal mouse cardiomyocytes (CMs) were blinded to isolate from 7-10 cases of postnatal 1-day old wildtype C57/B mice and cultured using Pierce Primary Cardi Page 10.omyocyte Isolation Kit (Thermo Fisher Scientific) as the manufacturer’s instructions. Cell passages 2-3 were used for the experiment. To induce hypertrophy, CMs were cpagesafd ultured in serum-free DMEM for 24 h and treated with 1 μM Angiotensin II (Sigma) for 48 h. Animal experiments were approved by the Animal Ethics Committee of the Second Affiliated Hospital of Wenzhou Medical University and conducted according to the Guid0e for the Care and Use of Laboratory Animals by the US National Institutes of Health.

Plasmids (2ug) or siRNAs (50 nM) were transfected into cells with Lipofectamine 3000 (Invitrogen, # L3000001) or Lipofectamine RNAiMAX (Invitrogen, #13778100) according to their manufacturer’s instructions, respectively. The short interfering RNAs (siRNA, 50 nM) including si-*Igf2bp2* (sc-146180), si-*HuR* (sc-35620), si-*Ythdf2* (sc-155424), si*-Fto* (sc-75003), si-*Ago2* (sc-44659), and si-RNA-A (sc-37007) were purchased from Santa Cruz Biotechnology. The plasmids for overexpression of IGF2BP2 (pcDNA3-GFP-IMP2-2, #42175) and control (pcDNA3.3_eGFP, #26822) were obtained from Addgene. The sequences of miR-133a inhibitor, mimics, and negative control (NC) were obtained from GEO, synthesized, and purified by GenePharma (Shanghai, China).

### Dual-luciferase reporter assay

The predicted binding sites of miR-133a on *Cdc42* and *Tead1* gene were obtained from online TargetScanMouse 7.2. miR-133a binding site region (WT) or mutant (MUT) on the *Cdc42* and *Tead1* 3’UTR was amplified and sub-cloned into a pmirGLO vector (Promega). When CMs were reached 70% confluence in 96-well plates, either reporter vector, miR-133a mimics, si-RNAs or NC were co-transfected using Lipofectamine 3000 for 48 h. Luminescent signal was measured using Dual-Glo Reagent (Promega) and taken by Luminometer (BMG LABTECH) following manufacturer’s instructions. The data were analyzed by calculating the ratio of luminescence from the experimental Renilla reporter to luminescence from the control Firefly reporter. The ratio was normalized to the ratio of control samples.

### Immunofluorescence (IF) staining

The treated CMs were fixed with 4% paraformaldehyde, permeabilized with 0.1% Triton X-100 in PBS, blocked with 3% BSA and incubated with cardiac troponin T (TNT) antibody (Thermo, # MA5-12960; 1:1000), m6A antibody (Synaptic Systems #202003, 1:1000), KI67 antibody (Abcam, #ab15580; 1:500) overnight at 4 °C. After washing, the cells were stained with fluorescence-conjugated secondary antibodies (Invitrogen; Alexa Fluor 488; #A32723 and Alexa Fluor 488; #A-11011, 1:500) and DAPI (Sigma, #9542). Immunofluorescence was analyzed with confocal microscopy FV1000MPE (Olympus Corporation), and the surface areas were measured using Image-J software [7, 45].

### Western blot analysis and co-immunoprecipitation (Co-IP)

As previous reports [46, 47], CM cells were lysed in radioimmunoprecipitation assay (RIPA) buffer and quantified by the Bradford method and Ponceau S staining of nitrocellulose membranes. 30 µg protein samples were subjected to 10% SDS-PAGE and transferred onto nitrocellulose membranes (Millipore). Immunoblot membranes were blocked in 5% milk for 1 h. Membranes were incubated with the primary antibody, including CDC42 (Santa Cruz, sc84011, 1:200), TEAD1 (Santa Cruz, sc393976, 1:200), AGO2 (Santa Cruz, sc376696, 1:500), IGF2BP2 (Proteintech, 11601-1-AP, 1:500) and GAPDH (Santa Cruz, sc-47724, 1:1000) at cold room for overnight. After three washes with TBST, membranes were incubated with secondary antibody for 1 h, washed, and exposed to Pierce ECL Western Blotting Substrate (Thermo, #32106) for detection of protein expression.

Proteins were lysed in RIPA buffer and immunoprecipitated with AGO2 or IGF2BP2 antibody and the corresponding IgG. After applying a magnet, proteins associated with Protein A/G Magnetic Beads were washed three times and analyzed by western blotting. The signal densities of protein bands were quantified and normalized to that of GAPDH using the ImageJ software.

### Quantitative real-time polymerase chain reaction (qRT-PCR)

Total RNA in CMs and ventricular was extracted using TRIzol reagent (Invitrogen, #15596026). For *Fto* and *Cdc42* mRNA assay, the first-stand cDNA was synthesized using TIANScript RT Kit (Tiangen, #KR104). qRT-PCR was performed with SYBR Green Master Mix (TaKaRa, #R820A). β-actin was used as an internal control. miR-133a and miR-499 mRNA was quantified using the Hairpin-it™ microRNA and U6 snRNA normalization RT-PCR quantitation kit (GenePharma). Relative changes in expression levels were calculated using the 2^−ΔΔCT^ method. All qRT-PCR analyses were performed in biological triplicates for each sample. The primers for m6A regulators, *Cdc42* were followed as previous report [48, 49]. The sequences of miR-133a, miR-499, and U6 were obtained from GEO.

### Measurement of mRNA m6A level

mRNA m6A level was detected by EpiQuik m6A RNA Methylation Quantification Kit (Colorimetric) (Epigentek). Total RNA in CMs and ventricular was extracted using TRIzol reagent and PolyA^+^ mRNA was purified using GenElute mRNA Miniprep Kit (Sigma-Aldrich). Then, mRNA m6A measurements were carried out according to the manufacturer’s instructions.

### m6A-mRNA Immunoprecipitation (m6A-RIP) qRT-PCR assay

Total RNAs were first extracted as above described. RNAs were treated with DNase to avoid DNA contaminations. RNA fragment was used according to mRNA Fragmentation Protocol (NEB, #E6110). Then, fragmented RNA was immunoprecipitated with m6A antibody (Synaptic Systems #202003) using EpiMark N6-Methyladenosine Enrichment Kit (NEB, #E1610S) following the manufacturer’s instructions. Enrichment of m6A containing mRNA was analyzed either through qRT-PCR as above described. The normal mouse IgG (Millipore. #CS200621) was used as IP control. 5% of the fragmented RNA was saved as input control. The related enrichment of m6A for individual mRNA was calculated by normalizing to 5% input.

### RNA stability assay

CMs were transfected sRNAs against *Igf2bp2, Ago2*, or Control for 24 h and then treated with 5 μg/ml actinomycin D for the indicated time. The total RNA was extracted by TRIzol reagent and analyzed by RT-PCR method.

### Statistical analysis

All analyses were blinded to perform with SPSS software version 25.0 (IBM Corp.) and GraphPad Prism 5 (GraphPad Software, Inc.). The numerical data are expressed as means ± standard deviation (SD) and obtained from at least in the triplicate experiment. Two-tailed *P* < 0.05 was considered statistically significant. Statistical significances were classified as *P < 0.05; **P < 0.01; ***P < 0.001. The comparison could be performed if the variance among groups is similar. Then, the independent-samples *t* test (unpaired) was used to compare two groups, and three or more groups comparison was involved ANOVA, followed by Bonferroni’s post hoc multiple comparison tests or Dunnett’s test.

## Supplementary information

Supplementary Figure Legends

Supplementary Table Legends

Supplementary Figure 1

Supplementary Figure 2

Supplementary Figure 3

Supplementary Figure 4

Supplementary Figure 5

Supplementary Figure 6

Supplementary Table 1

Supplementary Table 2

Supplementary Table 3

Supplementary Table 4
