# Supplementary material for: m6A modification promotes miR-133a repression during cardiac development and hypertrophy via IGF2BP2
Source: Cell Death Discov. 2021 Jun 26;7:157. doi: 10.1038/s41420-021-00552-7 (PMC8257704; doi:10.1038/s41420-021-00552-7)
Supplement: Supplementary file 1 — Supplementary Figure Legends [file 41420_2021_552_MOESM1_ESM.docx]

**Supplementary Figure legends**

**Figure S1. m6A modification associates cardiac miR-133a repression in heart development. A-B**, Heatmaps showing the mRNA expression of miR-133a and miR-499 target genes during heart development. Black square indicated the significantly down-regulated genes. **C**, The ratio of down-regulated targets of miR-133a and miR-499 between indicated stages. **D**, The percentages of down-regulated targets of miR-133a with m6A modification or not between indicated stages. E12.5, Embroy 12.5 day. P1, Postnatal day 1. 1m, 1 month.

**Figure S2. *Fto* responses to m6A modification during heart development. A**, Relative mRNA expression of m6A regulators in the adult heart (5 month). The *Fto* expression level was settled 1. **B**, Developmental expression profiles of m6A regulators in mouse embryonic and adult hearts. The expression change was calculated in relation to E14.5 data in B and D. Data are mean ± SD (n = 4 per stage). Data are mean ± SD. *P < 0.05; **P < 0.01, was determined by one-way ANOVA followed by Tukey’s test.

**Figure S3. Knockdown *Fto* increases the global m6A mRNA modification**. The primary cardiomyocytes were transfected of Si-*Fto* for 48h. Si-RNA-A was used as the transfection control. **A**, Successful decreasing of *Fto* mRNA expression by *Fto* knockdown using qRT-PCR analysis. **B**, Elevated m6A level by *Fto* knockdown using ELISA method. Data are mean ± SD (n = 4/group). P-value was calculated using the Student’s t-test.

**Figure S5. Heatmap and percentage of significant changes of miR-133a target proteins in the hypertrophic heart by TAC model.**

**Figure S6.** **IGF2BP2 binding to miR-133a targets with m6A modificattion**. **A-B**, Venn diagram indicating the overlap of IGF2BP2 binding genes and miR-133a (A) or miR-499 (B) target genes. **C**, The overlapping of the IGF2BP2 binding on miR-133a targets with or without m6A modification. **D**, The overall enrichment scores of miR-133a on the genes, with which binding of m6A and/or IGF2BP2 from the RISCome RNA-sequencing. Data are mean ± SD. P value was determined by one-way ANOVA followed by Tukey’s test.
