# Supplementary material for: m6A modification promotes miR-133a repression during cardiac development and hypertrophy via IGF2BP2
Source: Cell Death Discov. 2021 Jun 26;7:157. doi: 10.1038/s41420-021-00552-7 (PMC8257704; doi:10.1038/s41420-021-00552-7)
Supplement: Supplementary file 2 — Supplementary Table Legends [file 41420_2021_552_MOESM2_ESM.docx]

**Supplementary Table legends:**

**All supplementary tables are supplied as Excel datasheet.**

**Extend excel S1.** List of mouse cardiac miRNAs with or without m6A modification.

**Extend excel S2.** RISC-enrichment scores of miR-133a targets with or without m6A modification in miR-133a transgenic vs nontransgenic RISComes.

**Extend excel S3.** RISC-enrichment scores of miR-499 targets with or without m6A modification in miR-499 transgenic vs nontransgenic RISComes.

**Extend excel S4.** The overall enrichment scores of miR-133a target genes, with which binding of m6A and/or IGF2BP2 from the RISCome RNA-sequencing.
