# Supplementary figures and images for: m6A modification promotes miR-133a repression during cardiac development and hypertrophy via IGF2BP2
Source: Cell Death Discov. 2021 Jun 26;7:157. doi: 10.1038/s41420-021-00552-7 (PMC8257704; doi:10.1038/s41420-021-00552-7)

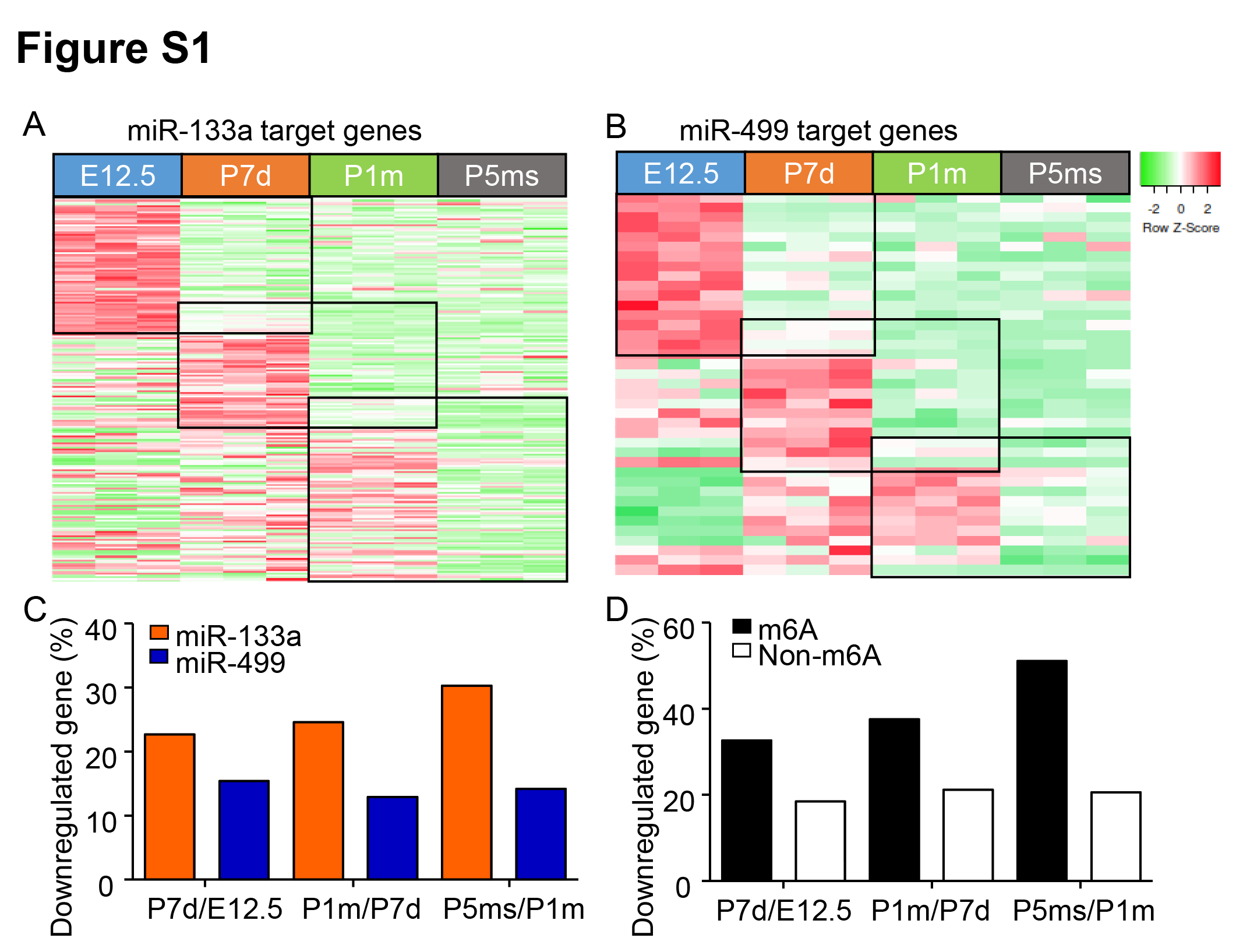

Supplement: Supplementary file 3 — Supplementary Figure 1 [file 41420_2021_552_MOESM3_ESM.tif]

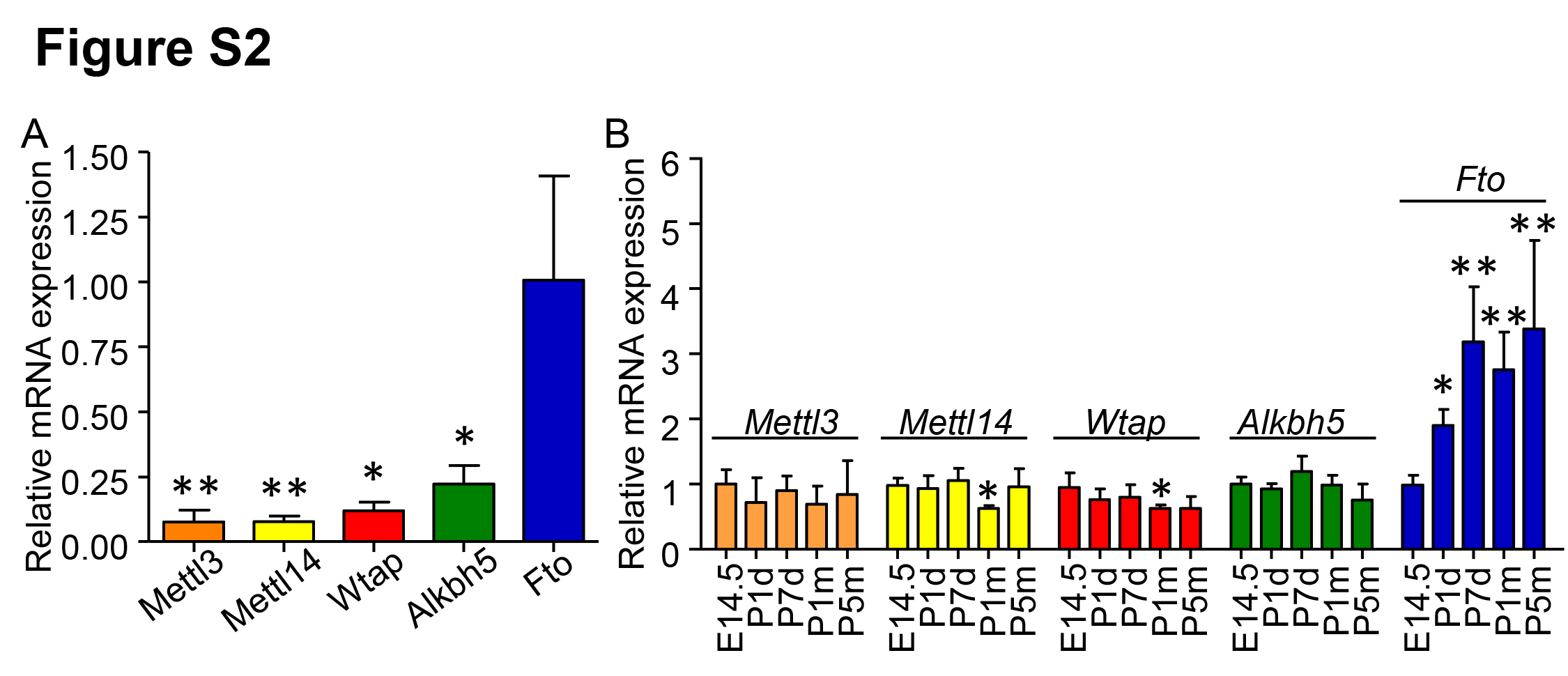

Supplement: Supplementary file 4 — Supplementary Figure 2 [file 41420_2021_552_MOESM4_ESM.tif]

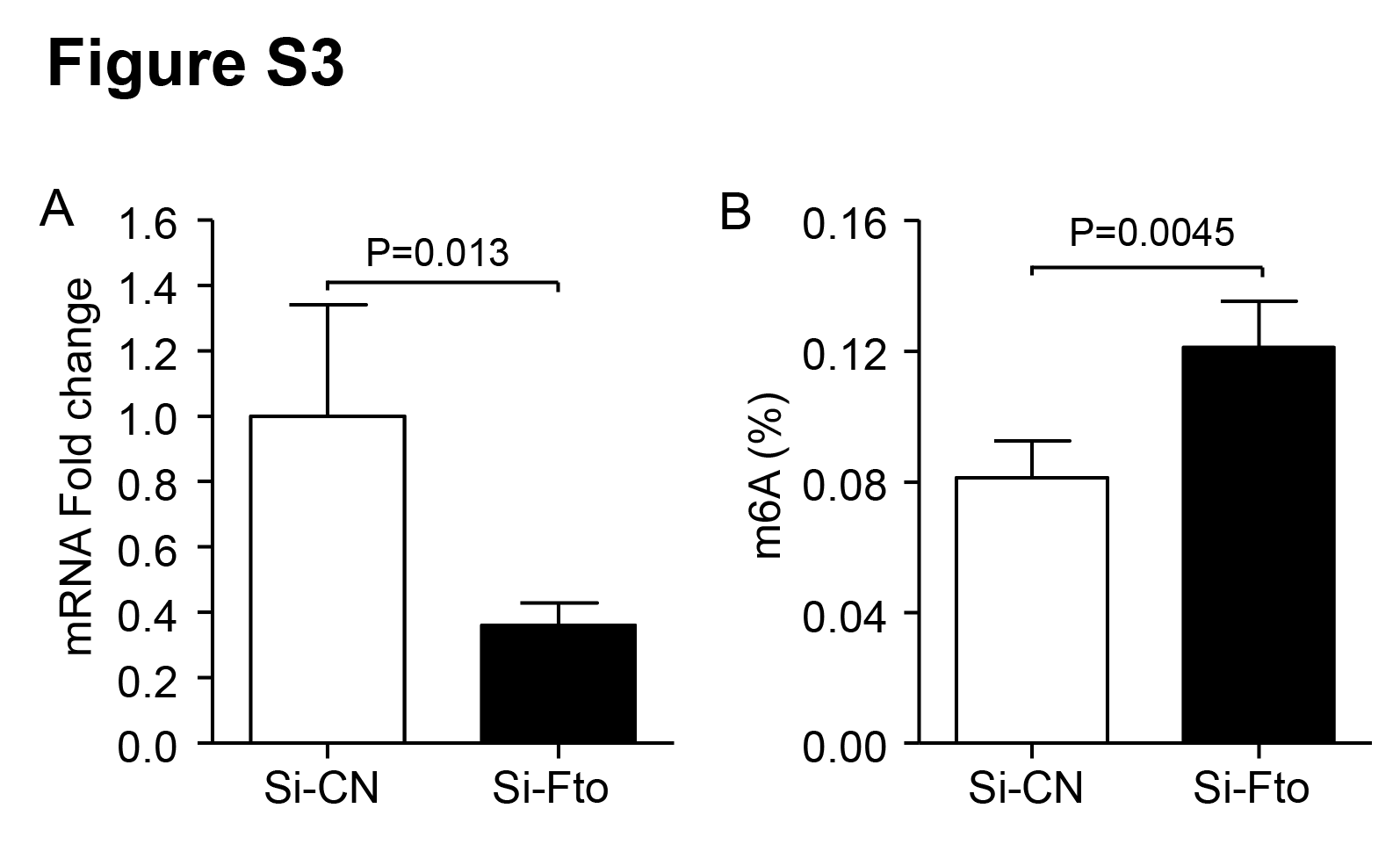

Supplement: Supplementary file 5 — Supplementary Figure 3 [file 41420_2021_552_MOESM5_ESM.tif]

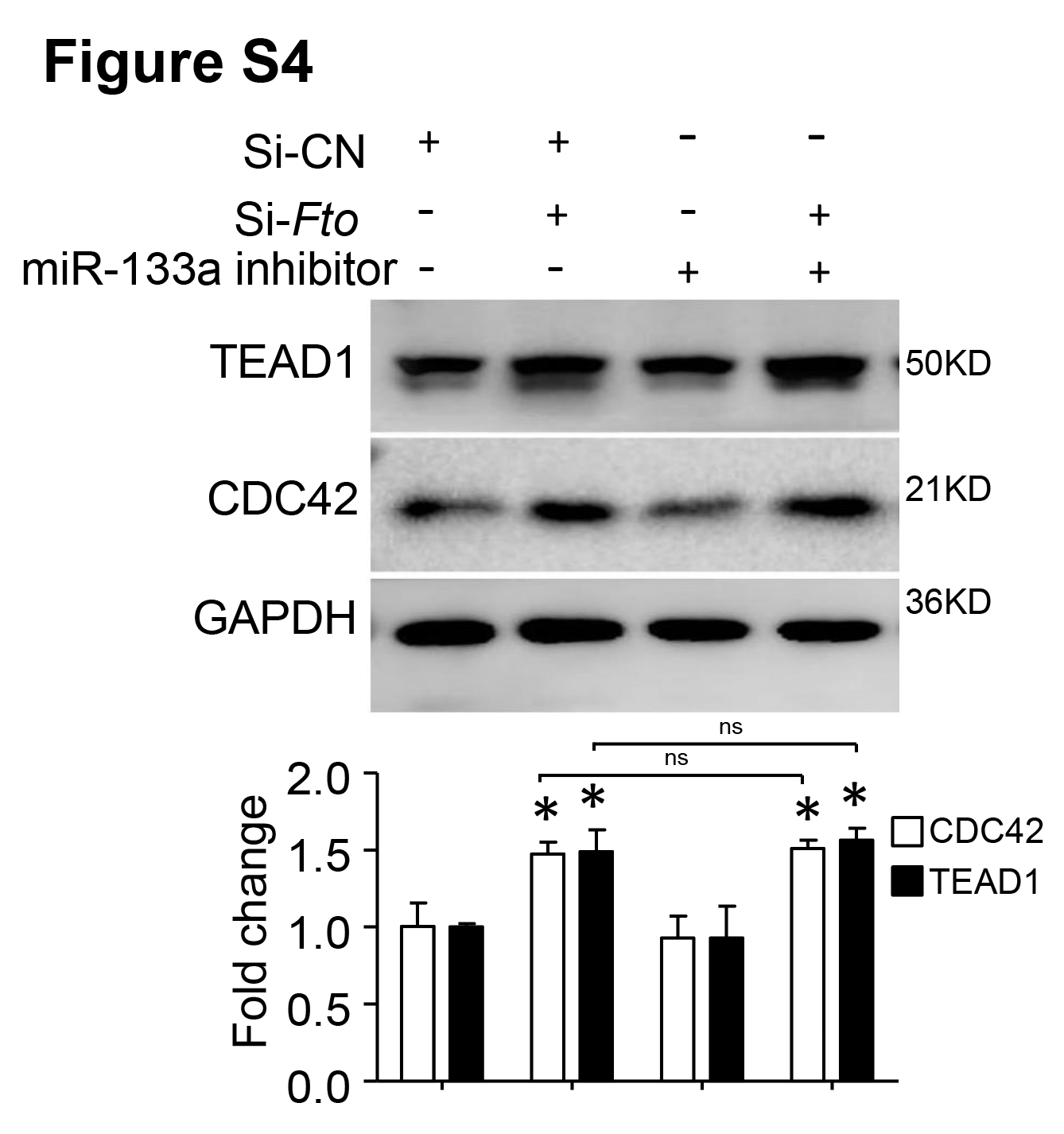

Supplement: Supplementary file 6 — Supplementary Figure 4 [file 41420_2021_552_MOESM6_ESM.tif]

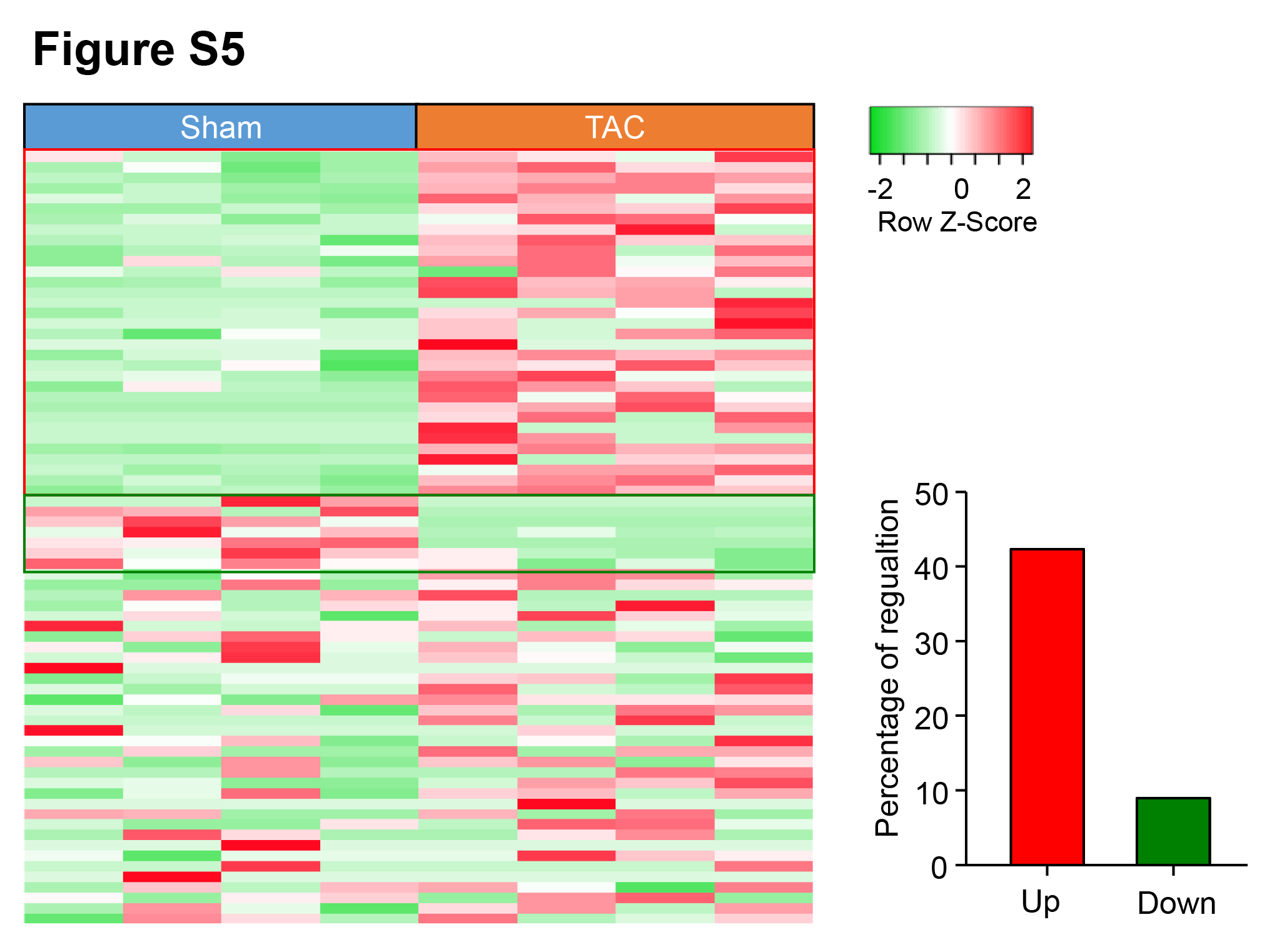

Supplement: Supplementary file 7 — Supplementary Figure 5 [file 41420_2021_552_MOESM7_ESM.tif]

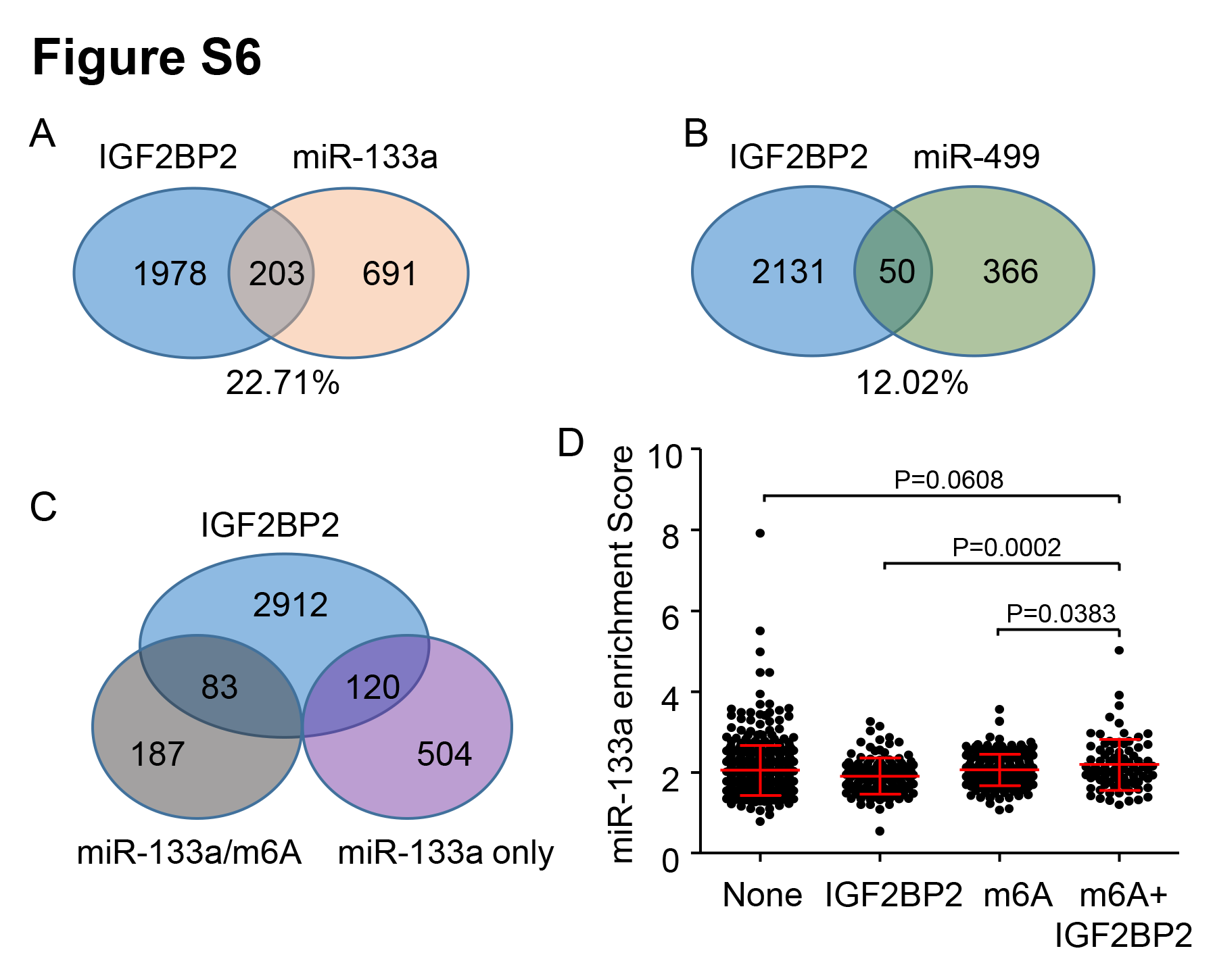

Supplement: Supplementary file 8 — Supplementary Figure 6 [file 41420_2021_552_MOESM8_ESM.tif]
